# Development and feasibility pilot study of positive psychological intervention for maintenance hemodialysis patients in China

**DOI:** 10.3389/fpsyg.2025.1693019

**Published:** 2025-10-20

**Authors:** Jie Yang, Yong-Qi Li, Xin-Yue Gao, Yong-Hui Cao, Jing Chen, Ling-ling Liu, Yi-Ru Wang, Yue-Qin Qian, Jing Wu, Jing Chu

**Affiliations:** ^1^Department of Nursing, Naval Medical University, Shanghai, China; ^2^School of Sociology and Political Science, Shanghai University, Shanghai, China; ^3^Ruijin Hospital, Shanghai Jiaotong University School of Medicine, Shanghai, China; ^4^Department of Nephrology, Shanghai Changzheng Hospital, Naval Medical University, Shanghai, China

**Keywords:** maintenance hemodialysis, psychological intervention, mental health, benefit finding, well-being, quality of life

## Abstract

**Background:**

Psychological problems are prevalent among maintenance hemodialysis (MHD) patients, and development of scientific, feasible and effective psychological promotion strategies can effectively improve their mental health. A series of studies were conducted to develop a group-based positive psychological intervention for MHD patients. The feasibility and effectiveness of the protocol were verified by a study.

**Methods:**

A cross-sectional survey of 246, including interviews with 13 patients, was conducted to determine the status and factors influencing mental health. A scoping review of 65 studies was performed to develop a protocol for the group-based positive psychological intervention for MHD was based on the PERMA theory and lasted for 4 weeks. Furthermore, experts, medical workers and patients who completed the intervention were interviewed to refine the program. Fifty-two patients were recruited from a hemodialysis center in Shanghai from December 2023 to January 2024. The primary outcome was benefit finding (BF). The secondary outcomes were well-being, quality of life (QoL), and the accessibility and implementation of the program, which were evaluated through interviews and researcher logs.

**Results:**

A total of 84 patients were recruited, 52 of whom ultimately participated in the study, including 26 patients in the intervention group and 26 patients in the control group. Twenty-four patients (92.31%) completed all interventions, and 26 patients (100%) completed more than 7 interventions. There were statistically significant differences in the intergroup effect (*F* = 4.603, *p* = 0.037 < 0.05), time effect (*F* = 5.663, *p* = 0.006 < 0.05) and interaction effect (*F* = 4.657, *p* = 0.015 < 0.05) of BF between the two groups. Moreover, through the intervention, the patients’ well-being and QoL in terms of the physical health dimension score improved.

**Conclusion:**

This study developed a group-based positive psychological intervention for MHD patients by conducting a scoping review, a cross-sectional study, qualitative interviews and expert group meetings. This study further optimized and verified the protocol of the intervention by analyzing the barriers to and facilitators of the intervention, conducting expert group meetings and performing the experiment. The intervention had a positive effect on physical and mental health for MHD patients.

**Clinical trial registration:**

Benefit Finding Promotion Program of Maintenance Hemodialysis Patients: An Empirical Study Based on the Implementation Science, identifier [https://www.chictr.org.cn/showproj.html?proj=209809, ChiCTR2300077469].

## 1 Introduction

The burden of chronic kidney disease (CKD) is increasing annually. It is associated with high disability rates, high medical costs and low awareness rates and has thus emerged as a serious global public health problem (GBD Chronic Kidney Disease Collaboration, 2020). According to the sixth China Chronic Disease and Risk Factor Surveillance, there were an estimated 82 million adults with CKD in mainland China from 2018 to 2019 (Wang et al., 2023). Maintenance hemodialysis (MHD) is the primary method of renal replacement therapy for individuals with kidney failure (Thurlow et al., 2021). Notably, China has one of the highest prevalence rates of MHD. According to the Chinese National Renal Data System (CNRDS), the number of registered individuals on MHD in China reached 7,844,265 by the end of 2022. As the number of MHD patients increases annually, the direct and indirect socioeconomic losses also become greater (Liang et al., 2024). Although dialysis can extend the life of patients, it is also characterized by a series of physiological and psychological challenges that require attention. Hemodialysis does not fully mimic the function of a normal healthy kidney; therefore, MHD patients may experience inappetence, nausea, vomiting, diarrhea, dry and itchy skin, limb edema, restless leg syndrome, joint pain, and sleep disorders (Raja and Seyoum, 2020; Scherer et al., 2017; Wang et al., 2024). Furthermore, MHD patients may experience changes in their body image, such as skin pigmentation, arteriovenous fistuloma formation, an oral ammonia smell, and facial puffiness (Johansen et al., 2021; Marthoenis et al., 2021; Wu et al., 2019). These symptoms may make patients reluctant to engage in social interactions, increase sensitivity and shame, and lead to perceived discrimination (He et al., 2022; Zheng et al., 2020). Previous studies have shown that most MHD patients suffer from depression, anxiety, sadness, and pain (Al-Shammari et al., 2021; Tian et al., 2021). These results suggest that there is an urgent need to attach importance to the psychological status of MHD patients.

Benefit Finding (BF) is as an important and novel concept in positive psychology (Helgeson et al., 2006), BF refers to the perception of an individual’s positive response to adverse life events, which manifests as positive cognitive and behavioral adaptation to adverse events (Sun et al., 2022; Yang et al., 2024a). BF can encompass a greater sense of personal strength, greater appreciation for life, enhanced relationships, spiritual growth, and new life opportunities (Aspinwall and Tedeschi, 2010). Furthermore, BF is considered an indicator of mental health (Yan et al., 2023). Previous studies have shown that higher levels of BF in individuals are correlated with higher levels of happiness and fewer negative emotions during illness (Wepf et al., 2022). Our research team has been committed to examining BF among MHD patients. Our studies have revealed that MHD patients have multiple BFs, including the search for meaning, gaining a sense of mastery, and self-enhancement (Gong et al., 2024) which was lower than that of older adults with chronic diseases (78.85 ± 16.70) (Zhang, 2018) and stroke patients (97.47 ± 17.64) (Wang, 2021) in China. BF was also found to be related to age, duration of HD, family support, other support, positive coping, and self-efficacy (Yang et al., 2024c). These findings provide a reference for the next step in the development of BF interventions.

Positive psychology has received considerable attention from the majority of researchers, thereby changing the status quo in psychological research, which had been dominated by negative psychology. Seligman proposed well-being theory, which comprises five elements of well-being, namely, positive emotion, engagement, positive relationships, meaning, and accomplishment (i.e., the PERMA theory) (Gander et al., 2016). This model focuses not only on medical issues but also on interpersonal relationships, interactions, communication, support from one’s surroundings, and personal values and achievements. In the context of positive psychology, the PERMA theory has achieved certain results in terms of improving the mental health of people, such as college students (Yang et al., 2024b) and breast cancer patients (Fang et al., 2023). This theory has been used to provide guidance to patients with respect to producing positive emotions during negative events. Therefore, the PERMA theory provides a basis for developing mental health intervention programs. The five elements of the PERMA theory enable researchers to observe and implement psychological interventions.

Therefore, on the basis of previous studies and guided by the PERMA theory, this study initially constructed a group-based positive psychological intervention for promoting BF in MHD patients and verified the scientific validity feasibility and effectiveness of the intervention to provide a theoretical and practical reference for future practice, to promote positive psychology in MHD patients and to improve mental health and QoL among MHD patients.

## 2 Methods

This study aimed to develop and validate a group-based positive psychological intervention for promoting BF in MHD patients. This study was conducted from March 2019 to January 2024 and included qualitative interviews, cross-sectional surveys, scoping reviews, interviews about barriers and facilitators factors for implementing the intervention, expert consultations, pretrials, and class trials. This study was approved by an Institutional Ethics Committee (Number: 20220715).

### 2.1 Development of the group-based positive psychological intervention for MHD patients

The development of the group-based positive psychological intervention comprised six sequential steps: (a) a comprehensive review of the literature to understand the conceptual connotation of BF; (b) a cross-sectional investigation in MHD patients to verify the level of BF and its influencing factors; (c) semistructured interviews with MHD patients to examine into the experience and external manifestations of BF; (d) a scoping review to summarize successful psychological interventions in previous studies; (e) the selection of intervention methods and strategies based on theory; (f) a demonstration through expert group meetings; and (g) a pretest to verify the clinical applicability and modify and improve the program.

Overall, the review of the literature suggested that BF is a widespread psychological experience among people with chronic diseases and that BF is affected by a variety of factors (Yang et al., 2024c). No consensus has been reached regarding the effect of demographic factors, such as age, education level, and economic income, on BF (Zimmaro et al., 2021). However, negative emotions such as anxiety and depression were found to be negatively correlated with BF (Mei et al., 2023; Zhu et al., 2022). Moreover, previous studies have reported that BF is positively correlated with social support (Qiu et al., 2022), positive coping (Li et al., 2023) and general self-efficacy (Helgeson et al., 2006) in patients. The BF of HD patients is lower than that of patients with other chronic diseases. Significant differences in BF scores were found between different age groups, HD duration categories, and degrees of HD-related knowledge. Taking BF as the dependent variable, the results of multiple linear regression analysis revealed that age, duration of HD, family support, other support, positive coping, and self-efficacy combined to explain 43.8% of the total variation.

The a semistructured interviews with MHD patients revealed that BF manifested in 3 ways (Gong et al., 2024): (a) the search for meaning, including approved hemodialysis, the desire to live; (b) gaining a sense of mastery, including adjusting self-psychology, developing healthy living habits, and learning hemodialysis-related behavior management; and (c) self-enhancement, including excavating external resources and affirming self-worth. These findings indicate that BF could help patients to develop a positive psychological framework by strengthening disease knowledge education, building a psychological mutual assistance platform, forming a multidisciplinary nursing team, providing effective social support resources, and cultivating patients’ self-health management. These approaches would improve the level and ability of benefit finding among MHD patients, help them to experience positive incentives, promote their physical and mental health, and improve their QoL.

Additionally, a scoping review was conducted to summarize mature and effective psychological interventions that promote BF based on the PERMA theory. The Embase, Cochrane Database of Systematic Reviews, PubMed, Web of Science, China National Knowledge Infrastructure, Sino-Med, Chongqing VIP, and Wan-fang databases were searched from inception to February 2023. We summarized and analyzed the relevant literature, including studies that examined psychological interventions to promote BF based on the PERMA theory. A total of 65 studies were included, and the results were as follows: (a) the goal of the interventions was to bring about long-term effects; (b) the interventions guided patients to reveal their true thoughts; (c) the interventions improved their ability to cope with negative events; (d) the form of the interventions was mainly offline, supplemented by online; (e) the themes of the interventions were simple and easy to understand; (f) the frequency of the interventions was usually 1–2 times per week; and (g) the outcome indicators focused on the positive factors influencing BF. Under the guidance of the PERMA theory, a group-based positive psychological intervention for promoting BF in MHD patients was initially developed, and 8 senior experts were invited to attend an expert meeting to refine the program.

**Figure 1:**
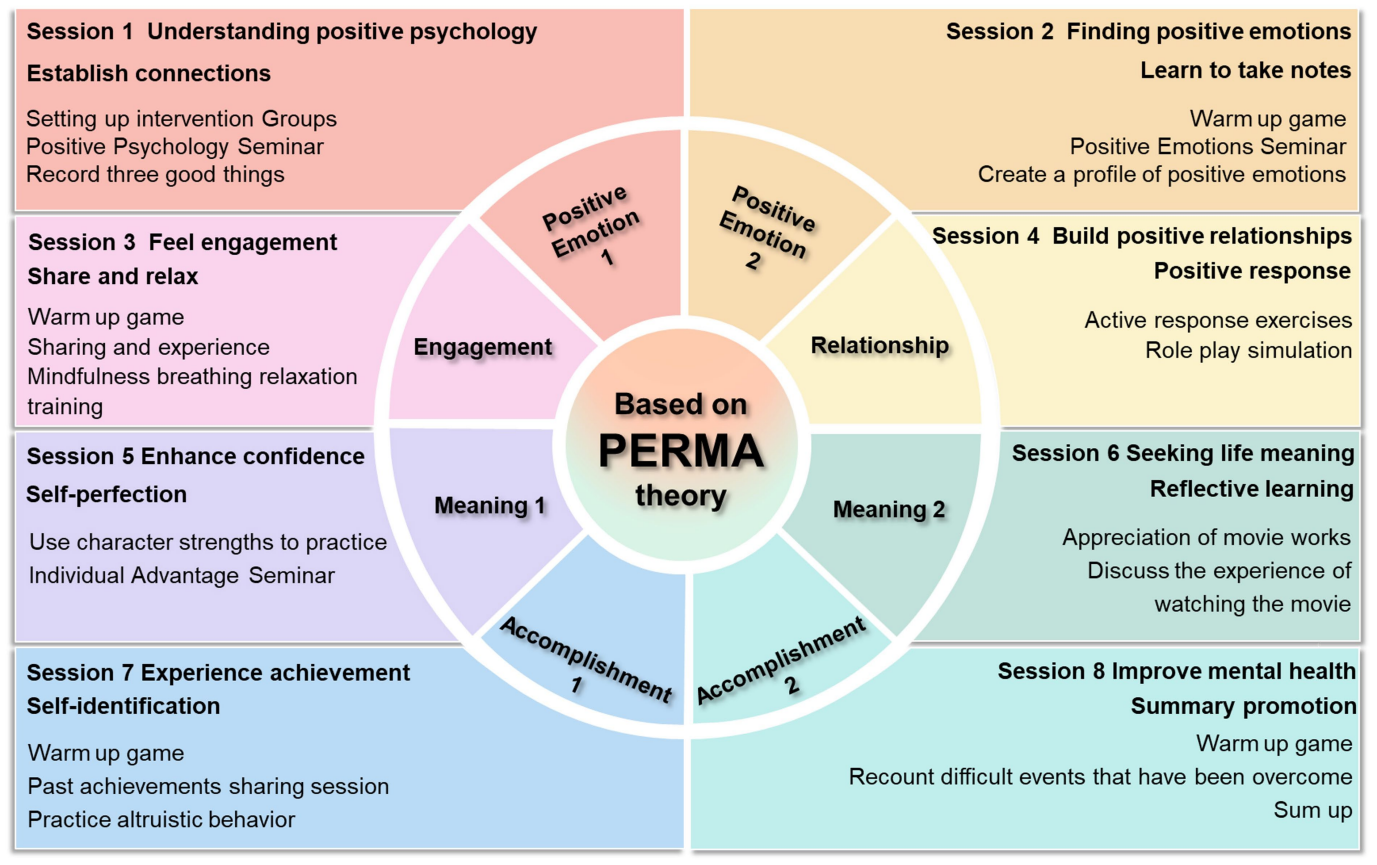
Group-based positive psychological intervention for MHD patients.

To further optimize the program, a descriptive qualitative research method was used to conduct semistructured interviews with stakeholders based on the i-PARIHS framework. Barriers to and facilitators of the application of the intervention in clinical practice were assessed and analyzed at 3 levels: *Innovation*, *Recipient*, and *Context* (inner context at local and organizational level and outer context at wider system and policy level) (Harvey and Kitson, 2016). A total of 21 stakeholders were interviewed; 11 barriers and 9 facilitators to the application of the program in clinical practice were analyzed. Countermeasures against these barriers were identified via literature review, group discussion and brainstorming (see Supplementary Material 1), and six experts were invited again to refine the intervention. Then, 6 MHD patients were tested to verify the feasibility, operability and acceptability of the group-based positive psychological intervention for promoting BF. Based on these findings, group programs can ultimately be implemented (see Figure 1).

### 2.2 Feasibility trial of the group-based positive psychological intervention for MHD patients

#### 2.2.1 Participants

Using a quasi-experimental study design, 52 MHD patients from a 3A hospital in Shanghai were selected as the study subjects from December 2023 to January 2024.

The inclusion criteria were as follows: (a) hemodialysis was performed as a kidney replacement treatment in line with the International Association of Nephrology ESRD diagnostic criteria (Webster et al., 2017); (b) age ≥18 years; (c) maintenance hemodialysis duration ≥3 months, with stable disease conditions; (d) clear consciousness, no cognitive impairment, and ability to communicate normally; (e) ability to independently use smartphones and the WeChat apps; and (f) signed informed consent forms and participated voluntarily. The exclusion criteria were as follows: (a) had undergone surgery recently; (b) had serious malignant diseases other than kidney disease; and (c) could not tolerate the intervention for approximately 1 h. The shedding criterion was participation in 8 interventions <6 times.

**Figure 2:**
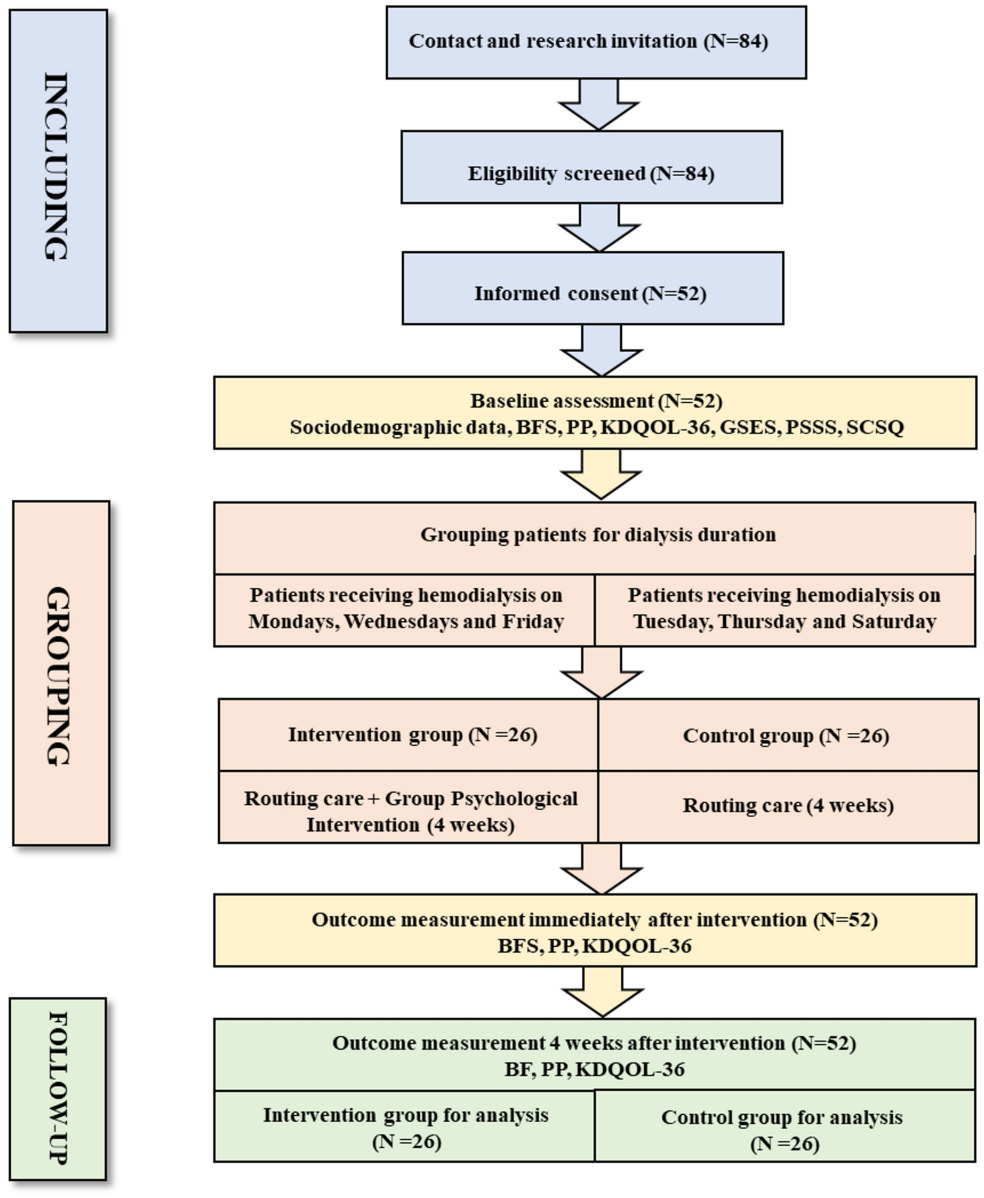
The flowchart for the inclusion of research participant.

Since MHD patients receive hemodialysis at a fixed time and frequency (three times a week, Monday, Wednesday, Friday or Tuesday, Thursday, and Saturday), a convenience sampling method was used, and patients receiving treatment on a single day of the week (Monday, Wednesday, and Friday) were included in the intervention group, while patients receiving treatment on other days were included in the control group to avoid contamination in the study (see Figure 2).

#### 2.2.2 Establish a professional intervention team

We established a multi-disciplinary intervention team that included individuals who have obtained certificates of competency in psychological counseling and services. Our research team and clinical staff assisted with the implementation of the intervention by helping with field control, participating in activities, demonstrating and introducing simulations, distributing and recycling intervention props, etc. Psychological experts supervised the entire process to control the quality of intervention. All team members were familiar with the content of the intervention program.

#### 2.2.3 Outcome measures

Informed consent was obtained from all individuals before the questionnaires were distributed. The main outcome measure was BF, and the secondary outcome measures were well-being, QoL, and general sociodemographic data. Coping styles, perceived social support, and self-efficacy were only measured at baseline. The psychometric assessment comprised different self-report measures: the Benefit Finding Scale (BFS) (Yan et al., 2023), the Kidney Disease Quality of Life scale (KDQOL-36) (Hays et al., 1994), the PERMA-Profiler (PP) (Yan, 2023), the Perceived Social Support Scale (PSSS) (Zhang et al., 2018), the General Self-efficacy Scale (GSES) (Wang et al., 2001), the Simplified Coping Style Questionnaire (SCSQ)(Xie, 1998). All the measures were completed at baseline (pre-intervention), postintervention, and 4 weeks after the intervention ended.

RE-AIM was used as the guiding framework to evaluate the application process of the program, including accessibility and implementation. Accessibility was evaluated by the participation rate, which was the proportion of participants who provided informed consent to participate in the study among the number of recruited people who met the inclusion criteria. The intervention included two dimensions: authenticity (interveners) and adherence (patients). Authenticity refers to the specific implementation of the intervention, such as whether it is implemented according to the original plan and whether the process is adjusted. Adherence refers to the percentage of patients who complete specific items of the intervention protocol.

#### 2.2.4 Intervention measures

The control group received standard care measures, including psychological support, guidance on dialysis-related knowledge, precautions during dialysis, dietary guidance, exercise guidance, and medication guidance, all provided by hemodialysis unit medical staff.

The intervention group received the group-based positive psychological intervention for MHD patients in addition to standard care. Patients were grouped in teams of 6–8 individuals, with interventions conducted once weekly on Mondays and Fridays. Each session lasted approximately 40 min over a 4-week period. Specific interventions included icebreaker games, themed group discussions, creating positive emotion portfolios, strengths-based exercises, role-playing, and other positive psychology practices. WeChat group chats facilitated information dissemination and supported intervention delivery, including: completing the online Strengths Finder assessment, summarizing intervention content, addressing patient inquiries and fostering communication.

#### 2.2.5 Data analysis

All analyses were performed via IBM SPSS software version 26 (IBM SPSS Inc. Chicago, Illinois, United States). The general demographic characteristics were described via counts and percentages. A *t*-test of two independent samples was used to compare groups at the same time, repeated-measures ANOVA was used to compare the overall change trend at different times, and one-way repeated-measures ANOVA was used to understand the change trend within different groups.

## 3 Results

### 3.1 Participant characteristics

A total of 52 questionnaires were sent out, and 52 were recovered, thus yielding an effective recovery rate of 100%. The results revealed no statistically significant difference in the baseline data between the two groups of MHD patients (*p* > 0.05) as shown in Tables 1, 2.

**Table 1:** Demographic characteristics of participants between two groups (*N* = 56).

Categorical variables	Intervention group (*n* = 26)		control group (*n* = 26)		*χ*^2^/*Z*	*P*
	*N*	%	*N*	%		
Gender						
Male	19	73.1	13	50.0	2.925^a^	0.087
Female	7	26.9	13	50.0		
Age						
18–30	1	3.8	0	0.0	−1.504^d^	0.133
31–40	5	19.2	4	15.4		
41–50	12	46.2	9	34.6		
51–60	5	19.2	6	23.1		
>60	3	11.5	7	26.9		
Marital status						
Married	19	73.1	23	88.5	4.139^c^	0.190
Unmarried	6	23.1	2	7.7		
Divorced	0	0.0	1	3.8		
Widowed	1	3.8	0	0.0		
Living style						
Live alone	2	7.7	2	7.7	1.080^c^	1.000
Live with family	23	88.5	24	92.3		
Other	1	3.8	0	0.0		
Caregiver						
Parents/children	6	23.1	10	38.5	5.815^a^	0.055
Companion	11	42.3	14	53.8		
In person	9	34.6	2	7.7		
Education						
Junior or lower	4	15.4	5	19.2	−1.195^d^	0.232
Senior high	3	11.5	8	30.8		
College	19	73.1	12	46.2		
Master degree or high	0	0.0	1	3.8		
Occupation						
Full-time	16	61.5	12	46.2	7.002^c^	0.068
Part-time	3	11.5	2	7.7		
Retire	3	11.5	11	42.3		
Unemployment	4	15.4	1	3.8		
Economic burden						
Very mild	4	15.4	5	19.2	−0.625^d^	0.532
Mild	8	30.8	10	38.5		
Moderate	8	30.8	5	19.2		
Serious	4	15.4	5	19.2		
Very serious	2	7.7	1	3.8		
Other chronic diseases						
Yes	11	42.3	14	53.8	0.693^a^	0.405
No	15	57.7	12	46.2		
Duration of MHD						
0–1 years (including 1 year)	3	11.5	4	15.4	−0.543^d^	0.587
1–5 years (including 5 year)	10	38.5	8	30.8		
5–10 years (including 10 year)	8	30.8	5	19.2		
≥10 years	5	19.2	9	34.6		
Complication						
Yes	6	23.1	7	26.9	0.103^a^	0.749
No	20	76.9	19	73.1		
Kidney transplantation						
Yes	3	11.5	3	11.5	0.000^b^	1.000
No	23	88.5	23	88.5		
Kidney transplantation intention						
None	9	34.6	10	38.5	−0.688^d^	0.491
General	10	38.5	12	46.2		
Vehemence	7	26.9	4	15.4		
Understanding degree of MHD						
Complete understanding	2	7.7	1	3.8	−0.955^d^	0.340
Adequate understanding	14	53.8	13	50.0		
Partial understanding	10	38.5	9	34.6		
No understanding	0	0.0	3	11.5		
Self-assessed MHD severity						
Very mild	1	3.8	0	0.0	−0.352^d^	0.725
Mild	4	15.4	5	19.2		
Moderate	13	50.0	15	57.7		
Serious	7	26.9	5	19.2		
Very serious	1	3.8	1	3.8		

^a^Pearson Chi-square test; ^b^continuity correction; ^c^Fisher exact test; ^d^Rank sum test.

**Table 2:** Psychological variables of participants between two groups (*N* = 56).

Variables	Intervention group (*n* = 26)	Control group (*n* = 26)	*t*	*P*
	( $\bar{x}\pm s$ )	( $\bar{x}\pm s$ )		
Positive coping	21.35 ± 6.54	20.73 ± 6.91	0.330	0.743
Negative coping	9.58 ± 5.43	9.27 ± 4.97	0.213	0.832
Self-efficacy	26.19 ± 6.21	27.35 ± 6.34	−0.663	0.511
Social support	55.85 ± 17.60	57.85 ± 13.39	−0.461	0.647

### 3.2 Acceptability and implementation of the interventions

#### 3.2.1 Accessibility

A total of 84 MHD patients who met the inclusion and exclusion criteria were recruited from November to December 2023, among whom 35 did not participate in the study. A total of 52 MHD patients ultimately participated in the study, with a participation rate of 61.9% (see Figure 2). During the recruitment of study subjects, we used research logs to record in detail the reasons for MHD patients’ reluctance to participate in the study. Through content analysis, the following four themes were summarized: (a) lack of motivation to participate; (b) difficulty in understanding the program; (c) doubtful effects of the intervention; and (d) restrictions on health status (see Supplementary Material 2).

#### 3.2.2 Implementation

During the implementation of this study, 24 patients (92.31%) completed all 8 interventions, and 26 patients (100%) completed more than 7 interventions. The group-based positive psychological intervention program was implemented two times each week for a total of 4 weeks and included a total of 21 specific interventions. Specifically, 20 interventions were successfully implemented in accordance with the original plan and rules. In the second intervention (finding positive emotions), some patients lacked interest in the warm-up game and were unwilling to participate. Therefore, the implementers adjusted according to the scene situation and moved on to the next step in advance (see Supplementary Material 3).

### 3.3 Effectiveness of the interventions

#### 3.3.1 Comparison of BF and well-being scores between the two groups

**Figure 3:**
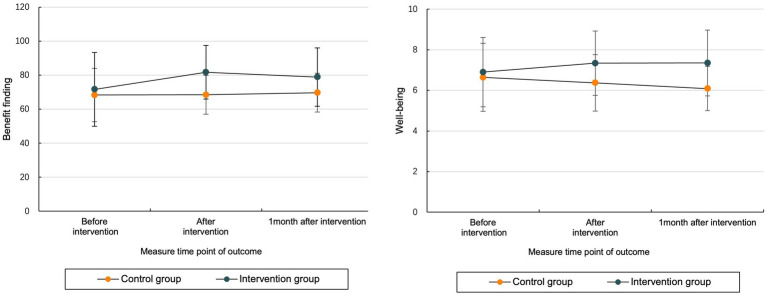
Comparison of BF and well-being scores at three intervention time.

The results of repeated-measures ANOVA revealed that there were interaction effects between intervention and time in BF and well-being scores in MHD patients, indicating that the magnitude of change in BF and well-being at three time points (before, after, and 4 weeks after intervention) over time was statistically significant (see Table 3 and Figure 3).

**Table 3:** Comparison of BF and PP scores at three intervention time.

Variable		BF		PP	
Groups		Intervention group	Control group	Intervention group	Control group
Pre-intervention		71.65 ± 21.66	68.35 ± 3.08	6.90 ± 1.71	6.64 ± 1.67
*t*		0.630		0.554	
*P*		0.531		0.582	
Post-intervention		81.73 ± 15.79	68.50 ± 11.40	7.34 ± 1.59	6.37 ± 1.39
*t*		3.463*		2.349*	
*P*		0.001		0.023	
4 weeks after intervention		78.88 ± 17.18	69.73 ± 11.42	7.35 ± 1.62	6.09 ± 1.09
*t*		2.263*		3.286*	
*P*		0.029		0.002	
Intergroup effect	*F*	4.603*		4.154*	
	*P*	0.037		0.047	
Intergroup effect	*F*	5.663*		0.989	
	*P*	0.006		0.376	
Interaction effect	*F*	4.657*		13.336**	
	*P*	0.015		<0.001	

* *= P* < 0.05, ** *= p* < 0.001.

#### 3.3.2 Comparison of QoL scores between the two groups

The results of the generalized estimation equation revealed that there were interaction effects between the intervention and time on the KDQOLTM-SF36 scores of MHD patients. The KDQOLTM-SF36 has five dimensions: physical health, mental health, symptoms and problems, kidney disease burden, and kidney disease impact. The results revealed that there was an intergroup effect on physical health between the two groups (*p* < 0.05), that there was a time effect on symptoms and problems, that there were effects on kidney disease and physical health (*p* < 0.05), and that there was no interaction effect in any dimension (*p* > 0.05) (see Table 4).

**Table 4:** Comparison of QoL at three intervention time.

Variable		Symptoms and problems		Kidney disease impact		Kidney disease burden		Physical health		Mental health	
Groups		IG	CG	IG	CG	IG	CG	IG	CG	IG	CG
Pre-intervention		76.60 ± 13.86	72.12 ± 20.95	56.85 ± 20.56	59.13 ± 20.12	42.79 ± 26.97	43.51 ± 25.65	37.87 ± 8.70	40.58 ± 9.29	51.98 (19.01)	42.09 (17.02)
*t/Z*		0.911		−0.952^a^		−0.099		−1.085		0.952^a^	
*P*		0.367		0.341		−0.099		0.283		0.341	
Post-intervention		85.42 (15.62)	83.33 (13.02)	62.40 ± 21.28	66.71 ± 15.69	50.24 ± 25.03	46.39 ± 30.53	38.14 (11.17)	46.45 (17.48)	53.45 (11.35)	49.10 (15.73)
*t/Z*		0.899^a^		0.899^a^		0.497		1.839^a^		1.592^a^	
*P*		0.369		0.369		0.622		0.066		0.111	
4 weeks after intervention		79.65 ± 12.94	81.01 ± 12.78	61.42 ± 18.79	69.83 ± 16.51	50.96 ± 25.35	47.92 ± 22.46	40.55 (10.60)	47.92 (15.17)	47.54 ± 9.21	49.15 ± 8.21
*t/Z*		−0.382		−1.715		0.458		2.260^a*^		−0.668	
*P*		0.704		0.093		0.649		0.024		0.507	
Intergroup effect	Wald *χ*^2^	0.334		1.288		0.112		3.959^*^		0.617	
	*P*	0.563		0.256		0.737		0.047		0.432	
Intergroup effect	Wald *χ*^2^	15.548**		12.039*		5.697		7.557*		3.756	
	*P*	<0.001		0.002		0.058		0.023		0.153	
Interaction effect	Wald *χ*^2^	5.229		2.135		0.720		1.313		5.851	
	*P*	0.073		0.344		0.698		0.519		0.054	

IG, intervention group; CG, control group. ^a^ = Z, * = *P* < 0.05, ** = *P* < 0.001.

## 4 Discussion

This study synthesized a variety of research methods to developed and validate a group-based positive psychological intervention for MHD patients in China. To our knowledge, this is the first study to promote BF in MHD patients by integrating the investigation, development and implementation of an intervention.

In the process of long-term hemodialysis, MHD patients face three kinds of physiological, psychological and social problems, namely, economic burden, depression, fatigue, weakness, pain, and sleep disorders (Huang, 2019; Ng et al., 2021), and they have strong needs for care and great psychological pressure (Ahmad and Al Nazly, 2015); thus, psychological intervention is urgently needed to improve their mental health. BF is an important concept in positive psychology, and numerous studies have demonstrated. BF is an important concept in positive psychology, and numerous studies have demonstrated this concept. With flourishing as the optimal psychological state, the PERMA theory has five elements that make the construction of intervention plans more observable and operational. Moreover, many previous studies have applied this theory to carry out group-based positive psychological interventions among college students (Yang et al., 2024b), patients with breast cancer (Fang et al., 2023) and Chinese adults (Nie et al., 2024), thus providing a valuable reference for the development of this research.

Previous studies on psychological interventions in MHD patients have focused more on improving negative emotions, whereas fewer studies have explored improving positive emotions. In fact, positive mental health is more conducive to physical rehabilitation, and its effect is more sensitive and obvious than that of alleviating negative emotions. The cross-sectional findings from this study revealed that the level of benefit found was lower in patients with MHD than in other patients with chronic diseases, which suggests that the factors influencing BF should be considered when providing patients with adequate emotional assistance and information, thus enhancing their self-efficacy, increasing perceived social support and improving positive coping skills with respect to dialysis-related problems. Furthermore, BF is likely to improve QoL by summarizing the mature and effective intervention measures in previous studies, such as character strength exercises, the establishment of positive emotion files, and the recording of three good things, The PERMA theory involves the rational use of five elements: positive emotions (experiencing hedonic emotions such as joy and cheerfulness); better engagement (feeling purpose and connection to one’s activities, e.g., experiencing absorption in tasks); positive relationships (feeling others’ support, being cared about, and being satisfied with one’s social relationships); meaning (experiencing a sense of purpose and being connected to something greater that exceeds oneself); and accomplishment (mastering difficulties and attaining goals and feeling a sense of pride) (Seligman, 2018). These factors are consistent with the results of previous cross-sectional surveys and qualitative interviews, and BF among MHD patients is strongly associated with PERMA’ five elements, which is a concrete manifestation of positive relationships. Patients with MHD tend to *search for meaning, gain mastery* and *self-enhancement,* which is what they find meaning and accomplishment (Gong et al., 2024). While previous studies have predominantly focused on the well-being outcomes of the PERMA model (Zhang et al., 2025), there is a lack of research on its deep integration with BF. This synergy can yield more applicable interventions and practical operational pathways, particularly for patients with MHD.

The results of the feasibility study show that accessibility was indicated by the participation rate of the study subjects recruited, with 61.9% of MHD patients willing to participate, indicating that most of the patients hoped to improve their mental health. Furthermore, 38.1% of the MHD patients failed to complete the intervention. The reasons for non-completion included a lack of motivation, difficulty in understanding, doubts about effectiveness, and limitations in health conditions. The compliance of the intervention group patients was high: 24 patients (92.31%) completed 8 interventions, and 2 patients completed the intervention 7 times (7.69%) because of personal reasons. In conclusion, the noninvasive psychological intervention developed herein was acceptable, operable and feasible. The program also has good extensibility; the fidelity of implementation was 95.24% (20/21), and only one intervention was not carried out. Through communication with MHD patients through feedback from the implementation of the program, after completing the intervention regarding mastery of psychological adjustment skills and methods and close communication with other patients, some patients were more prone to self-disclosure, some patients felt that their mental health had improved, and some patients hoped to continue participating in these interventions to consolidate the effect. It is recommended that the program continue to be promoted to obtain sustainable benefits.

The self-reported results of MHD patients revealed that BF and well-being effectively improved after the intervention, and the degree of improvement in the intervention group was significantly greater than that in the control group, which are similar as Liu (Liu et al., 2025). Furthermore, our results align with the established efficacy of PERMA-based models in other patient populations. For instance, such interventions have been shown to reduce fear and enhance psychological capital, overall well-being, and QoL in stroked patients (Luo et al., 2025), as well as alleviate fatigue and improve both QoL and psychological well-being in cancer patients (Hu et al., 2025). However, there was no significant effect on QoL. The main reason may be that the program focuses mainly on the expression of patients’ inner emotions, while it is not effective for health behaviors, especially those related to physical activity (such as work and limited activity). In addition, differences in the individual health level and cognitive style of patients may affect their subjective judgment of the impact and burden of kidney disease, and differences in disease severity and dialysis complications may also affect QoL (Dembowska et al., 2022). While some studies report an acceptable quality of life in MHD patients, factors such as number of complications, income, and years on dialysis are established determinants (AlRowaie et al., 2023; Yonata et al., 2022). Specifically, Complications like pruritus, sexual dysfunction, and physical limitations are strongly associated with a significantly poorer QoL (Alencar et al., 2020). Although these somatic and socioeconomic factors are not easily modifiable through brief psychological interventions, the efficacy of such interventions in improving QoL is well-documented (Yan et al., 2025). Therefore, personalized interventions can be designed to improve the QoL of MHD patients by accounting for individual differences and external environmental factors.

### 4.1 Limitations

This study is the first application of a group-based positive psychological intervention for promoting BF in MHD patients. The sample size was small, and it was a single-center, quasi-experimental study. More high-quality, large-sample, multicenter experimental studies are needed in the future to further clarify the application effect of the program and further improve the program content and optimize the implementation process so that the program can be promoted and applied in clinical practice. Additionally, this study’s findings are influenced by its cultural context. Given our focus on a mainland Chinese population, the applicability of the results to other communities may be limited due to cultural differences.

## 5 Conclusion

The MHD patients are affected by factors such as disease and long-term treatment and often face heavy physical and mental health burdens. The status and prognosis of CKD cannot easy be changed for most MHD patients. Therefore, changing their understanding of the disease, discovering the benefits of dialysis, and seeking ways to coexist with dialysis are effective strategies to reduce negative psychological reactions, promote the production of positive emotions, and maintain mental health. On the basis of the literature review, cross-sectional investigation, qualitative interviews and expert group meetings, this study developed a group-based positive psychological intervention for promoting BF in MHD patients and further optimized and verified the final version of the program through interviews with promoting and hindering factors, expert group meetings and a feasibility study. The program has clinical application and promotion value, which can effectively improve the level of BF and well-being and improve the physical and mental health of MHD patients.

## Data Availability

The raw data supporting the conclusions of this article will be made available by the authors, without undue reservation.

## Supplementary material

SUPPLEMENTARY MATERIAL 1 Barriers and facilitators based on the i-PARIHS framework to promote the BF of MHD patients.

SUPPLEMENTARY MATERIAL 2 Reasons for MHD patients not participating in the study.

SUPPLEMENTARY MATERIAL 3 Barriers, facilitators factors and countermeasures in the implementation of intervention program.
