# Supplementary material for: Development and feasibility pilot study of positive psychological intervention for maintenance hemodialysis patients in China
Source: Front Psychol. 2025 Oct 20;16:1693019. doi: 10.3389/fpsyg.2025.1693019 (PMC12580280; doi:10.3389/fpsyg.2025.1693019)
Supplement: Supplementary file 1 [file Supplementary_file_1.docx]

**Supplementary 1**

Objective sampling method was used to select medical staff and MHD patients in hemodialysis room of a three A hospital in China from March to May 2023. Determine the sample size in a pattern of deductive topic saturation, and add 1-2 more samples to ensure that no new information emerges.

**Table 1 General information of medical staff**

| **No.** | **Gender** | **Age** | **Education** | **Professional title** | **Working years** | **Participated in psychological training** |
| --- | --- | --- | --- | --- | --- | --- |
| N1 | Female | 52 | Junior college | Intermediate | 33 | NO |
| N2 | Female | 22 | Junior college | Primary | 2 | YES |
| N3 | Female | 50 | Undergraduate | Deputy chief | 30 | YES |
| N4 | Female | 29 | Undergraduate | Primary | 6 | NO |
| N5 | Female | 51 | Junior college | Primary | 32 | YES |
| N6 | Female | 35 | Undergraduate | Intermediate | 13 | NO |
| N7 | Female | 23 | Junior college | Primary | 1 | NO |
| N8 | Male | 22 | Junior college | Primary | 2 | YES |
| N9 | Female | 52 | Junior college | Intermediate | 30 | YES |
| N10 | Female | 40 | Undergraduate | Intermediate | 20 | YES |
| D1 | Female | 37 | Master's degree | Attending physician | 2 | YES |
| D2 | Female | 35 | Doctor's degree | Attending physician | 3 | NO |

N = Nurse, D = Doctor

**Table 2 General information of maintenance hemodialysis patients**

| **No.** | **Gender** | **Age** | **Marital status** | **Caregiver** | **Education** | **Duration of hemodialysis** | **Participated in psychological training** |
| --- | --- | --- | --- | --- | --- | --- | --- |
| P1 | Female | 60 | Married | Companion | Senior high | 15 | NO |
| P2 | Male | 67 | Married | Companion | College | 14 | NO |
| P3 | Male | 42 | Unmarried | In person | College | 20 | NO |
| P4 | Male | 68 | Married | Companion | Junior | 22 | NO |
| P5 | Female | 44 | Unmarried | In person | College | 3 | NO |
| P6 | Male | 53 | Married | Companion | Junior | 8 | NO |
| P7 | Female | 46 | Unmarried | Parents | College | 21 | YES |
| P8 | Male | 57 | Married | Companion | College | 10 | YES |
| P9 | Female | 40 | Married | Companion | College | 5 | NO |

Under the guidance of the i-PARIHS framework and with reference to its obstacle identification and evaluation list, and 11 barriers and 9 facilitators to the application of the program in clinical practice for the benefit finding promotion program of maintenance hemodialysis patients were extracted, then corresponding countermeasures were drawn up based on the methods of literature review, group discussion and brainstorming.

**Table 3 Barriers and facilitators based on the i-PARIHS framework to promote the benefit finding of maintenance hemodialysis patients**

| **Hierarchy** | | **Barriers** | **Facilitators** | **Countermeasure of Barriers** |
| --- | --- | --- | --- | --- |
| Innovation | | a. Some psychological intervention measures are not specific and difficult to implement directly.  b. Some psychological intervention measures are highly professional and difficult to implement.  c. Some psychological interventions have cultural differences.  d. The implementation period of psychological intervention program is long, and follow-up is challenging.  e. It is difficult to implement and promote group psychological intervention. | a. The psychological intervention program was derived from high-quality studies with reliable content.  b. The psychological intervention program is rich in content, diverse in form and highly interactive. | a. Further refine and optimize psychological intervention programs to form guidance manuals that can be directly implemented.  b. Further adjust the specific content of the program through pre-experiments.  c. The modified plan was to continue for 4 weeks, twice a week, and conduct 1-month follow-up.  d. Strengthen communication with patients and establish a sound data collection and follow-up mechanism. |
| Recipients | Team | The ability of team members to implement innovation is insufficient. | a. The establishment of the university-hospital research cooperation team improved the feasibility.  b. Ward leaders strongly supported the implementation of reform. | Integrate resources, give full play to their respective advantages, and strengthen multidisciplinary team building and cooperation. |
|  | Individual  (Medical staff) | a. Some individuals are not familiar with the content of psychological intervention program.  b. Some individuals are not qualified for psychological intervention. | a. The individual has a higher understanding of the patient.  b. Individuals have a high sense of identity and strong willingness to participate in the innovation.  c. Individual have a spirit of dedication. | a. Provide training and explanation to relevant individuals.  b. Improve individuals' understanding of mental health interventions through lectures. |
|  | Individual  (Patients) | a. Some patients have doubts.  b. Some patients aren’t motivated to participate.  c. The acceptability of patients varies.  Simple psychological intervention does not meet the needs of patients. | a. High individual enthusiasm.  b. Some patients have relevant experience. | a. Expand online and offline multi-channel publicity.  b. Explain and communicate well with family members and patients.  c. Increase the content of disease knowledge popularization. |
| Context | Inner context  (Local) | a. Lack of a certain incentive mechanism.  b. Lack of a positive climate suitable for implementing changes.  c. Lack of suitable intervention sites. | a. The ward has certain experience in implementing activities.  b. The ward has certain psychological nursing measures, and achieved good results.  c. The ward has an independent public number for patients to obtain relevant information. | a. Prepare small gifts for subjects.  b. Provide appropriate incentive mechanisms for intervention implementers. |
|  | Organization | Lack the successful experience and case of innovation. | a. The hospital is one of the largest hemodialysis centers in Shanghai, providing security for the participants.  b. The staff in the ward is relatively stable, which is easy to follow up. | Through literature review and qualitative interview, learn and understand the successful experience of relevant innovation. |
|  | Outer context  (Wider system and policy level) | a. Lack of norms and standards for psychological intervention.  b. Lack of extensive science education of social psychological knowledge. | 1. The direction of innovation conforms to the national policy.  2. Technological progress and policy improvement. | Formulate intervention rules and strictly implement intervention measures. |

**Supplementary 2**

**Table 4 Reasons for MHD patients not participating in the study**

| **Them** | **The patient's words** |
| --- | --- |
| Lack of motivation to participate | P1: I think I'm pretty sane, and I don't need this kind of intervention.  P2: Ah, I don't have any psychological problems. Why would I participate in such a study? |
| Difficulty in understanding the program | P3: I've been trying to figure out what you're doing.  P4: Why do we have to do this so many times? Can I just be part of this.  P5: I don't know how to cooperate with all the interventions you're talking about? |
| Doubtful Effect of intervention | P6: Will doing these things make my feel better, improve the quality of my life? Not necessarily.  P7: I think some of the content of your program is too simple. It sounds like a game for children. |
| Restrictions on health status | P8: I am so weak with this disease that I don't want to do any more superfluous things.  P9: I don't have the energy to do so many activities, you can find those who are in better condition. |

P = Patients

**Supplementary 3**

**Table 5 Barriers, facilitators factors and countermeasures in the implementation of intervention program**

| **facilitators factors** | | **barriers factors** | | **countermeasures** |
| --- | --- | --- | --- | --- |
| **Theme** | **Specific elaboration** | **Theme** | **Specific elaboration** |  |
| The intervention content of the scheme is specific and detailed, with high feasibility. | The program has formulated specific rules, clarified the specific intervention content, form, duration, etc., and can be smoothly implemented. | Patient cooperation is limited | Some patients have low interest in some intervention measures and low participation enthusiasm. | Intervention team members took the initiative to communicate with patients, understand their true thoughts and suggestions, and timely adjust the program. |
| Clear division of responsibility among intervention team members | Each member of the intervention team is responsible for interfacing with the fixed patient and is responsible for the implementation of interventions in different parts of the group psychological intervention program. | The coverage of interventions is uncertain | Some patients may miss the key content of the intervention because of distraction, and each patient has uneven speaking time. | Increasing the number of intervention teams to ensure each patient could be concerned; Repeatedly strengthen the core content of intervention; Actively guide each patient to actively engage in group discussion. |
| Established a stable and close relationship with the patient | During the implementation of the intervention, the team members actively communicated with the patients and answered questions in time |  |  |  |
